# Natural course of care dependency in residents of long-term care facilities: prospective follow-up study

**DOI:** 10.1186/1471-2318-14-67

**Published:** 2014-05-22

**Authors:** Monique AA Caljouw, Herman JM Cools, Jacobijn Gussekloo

**Affiliations:** 1 Department of Public Health and Primary Care, Leiden University Medical Center, P.O. Box 9600, Leiden 2300 RC, the Netherlands

**Keywords:** Care dependency, Predictive factors, Variability, Mortality, Long-term care facility, Vulnerable older persons

## Abstract

**Background:**

Insight in the natural course of care dependency of vulnerable older persons in long-term care facilities (LTCF) is essential to organize and optimize individual tailored care. We examined changes in care dependency in LTCF residents over two 6-month periods, explored the possible predictive factors of change and the effect of care dependency on mortality.

**Methods:**

A prospective follow-up study in 21 Dutch long-term care facilities. 890 LTCF residents, median age 84 (Interquartile range 79–88) years participated. At baseline, 6 and 12 months, care dependency was assessed by the nursing staff with the Care Dependency Scale (CDS), range 15–75 points. Since the median CDS score differed between men and women (47.5 vs. 43.0, *P* = 0.013), CDS groups (low, middle and high) were based on gender-specific 33% of CDS scores at baseline and 6 months.

**Results:**

At baseline, the CDS groups differed in median length of stay on the ward, urine incontinence and dementia (all *P* < 0.001); participants in the low CDS group stayed longer, had more frequent urine incontinence and more dementia. They had also the highest mortality rate (log rank 32.2; df = 2; *P for trend* <0.001). Per point lower in CDS score, the mortality risk increased with 2% (95% CI 1%-3%). Adjustment for age, gender, cranberry use, LTCF, length of stay, comorbidity and dementia showed similar results. A one point decrease in CDS score between 0 and 6 months was related to an increased mortality risk of 4% (95% CI 3%-6%).

At the 6-month follow-up, 10% improved to a higher CDS group, 65% were in the same, and 25% had deteriorated to a lower CDS group; a similar pattern emerged at 12-month follow-up. Gender, age, urine incontinence, dementia, cancer and baseline care dependency status, predicted an increase in care dependency over time.

**Conclusion:**

The majority of residents were stable in their care dependency status over two subsequent 6-month periods. Highly care dependent residents showed an increased mortality risk. Awareness of the natural course of care dependency is essential to residents and their formal and informal caregivers when considering therapeutic and end-of-life care options.

## Background

The proportion of older people is steadily rising worldwide, people live longer and are managing their daily activities for longer than ever before [1]. But they have also a higher risk on negative health outcomes, like care dependency, being institutionalized and mortality [2,3]. Many vulnerable older people, heavily dependent on care, are living in long-term care facilities (LTCF) and place considerable constraints on healthcare professionals and healthcare budgets.

In the Netherlands 0.4% of the population; and around 2.7% of the population aged 65 years and above are living in LTCFs [4,5]. A typical Dutch LTCF accommodates 150–200 residents, has specialized psycho-geriatric wards for residents with dementia, somatic wards for residents with physical problems, and wards for rehabilitation [6].

The daily nursing care in LTCF focuses on residents’ care dependency as a process in which the residents’ self-care decreases, and in which care demands make a person increasingly dependent on nursing care [7]. However, care dependency behaves like a dynamic process that is influenced by illness and disability [8-10], i.e. care dependency can be a temporary, long-term or a permanent state [11].

Two recent studies investigated the natural course of activities in daily living (ADL) among nursing home residents. Both studies showed that residents could improve, be stable or deteriorate in their ADL performance during 6 months of follow-up [12,13]. A study in a selected population of 68 females with Alzheimer’s disease, living in a single Dutch LTCF and who survived a two-year period, care dependency showed a significant increase within that two-year period [14].

Previous studies have shown that i.e. nutritional status [12,13], cognitive impairment [12,13,15], absence of daily contact with proxies [12], depression [12,16], neuropsychological deficits [17], incontinence [12,13,18] and infections [19] were mentioned as predictors for deterioration in ADL performance of vulnerable older people. Deterioration in ADL will lead to more individual care demands and higher care dependency.

However, little is known about the natural course of care dependency in institutionalized older persons. It seems relevant to gain more insight in the stability and changes in care dependency to manage care and to provide better tailored care for individual LTCF residents. Therefore, we examined the changes in care dependency in LTCF residents over two 6-month periods, explored the possible predictive factors of change in care dependency, and the effect of care dependency on mortality.

## Methods

### Setting and study population

The present prospective follow-up study was conducted within the framework of the CRANBERRY trial. The Cranberry trial is a double-blind randomized placebo-controlled multi-center trial, in which a total of 21 LTCFs from the University Nursing Home Research Network in South-Holland, the Netherlands, participated (trial registration NTR1266). The CRANBERRY study assesses the effectiveness of cranberry capsules to prevent urinary tract infections in vulnerable older persons living in intramural care settings in which care for the most vulnerable older persons is provided by a multidisciplinary team including elderly-care physicians, nursing assistants, licensed practical nurses, registered nurses and paramedical professionals. Residents aged 65 years and over were included. Excluded were residents with a life expectancy shorter than 1 month or using coumarin. For detailed information on the study design and outcomes we refer to the publication of the original trial [20].

The Medical Ethics Committee of the Leiden University Medical Center approved the study. Written informed consent was obtained from all participants. For participants incapable of giving informed consent due to cognitive impairment, a guardian provided written consent.

### Care dependency

At baseline, and at 6 and 12 months follow-up, an assessment was made of the care dependency status by interviewing the responsible nurses who care for the participants. For this the Care Dependency Scale (CDS) was used, which is a tool completed by nursing staff for assessment of the care dependency status of institutionalized residents [21]. The CDS has satisfactory reliability and validity [22-24], and consists of 15 items, measuring basic care needs on a 5-point scale. The total CDS score ranges from 15 (completely dependent on care) to 75 (almost independent of care). The CDS 15 items are eating and drinking, continence, body posture, mobility, day and night pattern, getting (un)dressed, body temperature, hygiene, avoidance of danger, communication, contact with others, sense of rules and values, daily activities, recreational activities and learning ability.

Since women and men differ in their baseline care dependency status and the CDS scores were not normally distributed, women and men were separately ranked into gender-specific 33% groups according to their baseline CDS score. Thereafter, we combined the lowest, middle and highest 33% for women and men, to generate three gender-specific CDS groups. The ‘low score’ CDS group indicates participants most dependent on care and the ‘high score’ CDS group indicates participants the most independent of care.

### Patient characteristics

#### Socio demographic factors

At baseline, a research nurse collected information on the participants’ gender, age and length of stay on the ward.

#### Comorbidity

Information on participants’ medical history was obtained by examination of the medical records, and interviews with the elderly care physician. Within the CRANBERRY trial we obtained clinical information on the presence of myocardial infarction, stroke, cancer, diabetes mellitus, chronic pulmonary disease (COPD) and dementia, as well as information on urine incontinence and urinary tract infections in the preceding year.

### Statistical analysis

Comparisons were made between the CDS groups using Chi-square tests in case of categorical data and Kruskal-Wallis tests to compare the three groups for non-normally distributed continuous variables. *P*-values < 0.05 were considered significant and should be interpreted as nominal ones.

The difference in the cumulative incidence of mortality between the CDS groups was explored with Kaplan-Meier curves, with corresponding log-rank test. Cox proportional hazards models, adjusted for age, gender, cranberry use, LTCF, length of stay on the ward, somatic comorbidity (myocardial infarction, stroke, cancer, diabetes mellitus, COPD, urine incontinence, and urinary tract infection in the preceding year) and dementia were used to present mortality risks based on continuous CDS score at baseline.

The change in care dependency for survivors between 0–6 months and 7–12 months is presented by the number of participants in the three CDS groups who improved, stayed stable, or degraded to another CDS group during the two 6-month periods. For the analysis of CDS change in the subsequent 7–12 months, participants were newly classified in gender-specific 33% groups at the 6-month CDS assessment.

A crude and adjusted multivariate linear regression analysis was performed to estimate the predicted CDS score for survivors at 6-month follow-up. The CDS score at 6-month follow-up was considered as a dependent variable, while gender, age, cranberry use, LTCF, length of stay on the ward, CDS score at baseline, somatic comorbidity (myocardial infarction, stroke, cancer, diabetes mellitus, COPD, urine incontinence, and urinary tract infection in the preceding year) and dementia were considered to be independent variables. Except gender and age, all other variables with a *P*-value ≥ 0.05 were excluded from the adjusted model. Co-linearity between the independent variables and dependent variable (CDS score at 6 months) will be investigated with the Variance Inflation Factor (VIF). A VIF of 5 or above indicates co-linearity.

Analyses were performed with IBM SPSS Statistics for Windows, version 20.0.

## Results

In the original trial, 928 residents were included [20]. In 38 participants the baseline CDS score was missing due to technical reasons, resulting in a total of 890 participants eligible for the present study. There were no differences is gender, age and comorbidity between the participants and the 38 non-participants.

At 6 months follow-up, 132 participants (14.8%) had died and in 44 participants (4.9%) the CDS scores were missing, resulting in 714 participants (80.2%) at 6 months. At 12 months follow-up, another 129 participants (18.1%) died and in 21 participants (2.9%) the CDS scores were missing, resulting in 564 participants with complete measurements (79.0%) at 12 months.

### Study population

Table 1 presents the baseline characteristics of the total population and of the three CDS groups. Overall, almost 75% of the study population was female and the median age was 84 (IQR 79–88) years. The median CDS score was 44 (IQR 30–56). At baseline, women had a lower CDS score compared with men: 43 (33rd percentile 34, 66th percentile 51) vs. 47.5 (33rd percentile 37, 66th percentile 55); Mann–Whitney U-test; *P* = 0.013.

**Table 1:** Baseline characteristics of the total study population and the three care dependency groups based on their care dependency scores at baseline

		**Care dependency groups**^**#**^			
	**Total population**	**Low CDS group**	**Middle CDS group**	**High CDS group**	
	**n = 890**	**n = 303**	**n = 282**	**n = 305**	*P*-value*
*Cut-off level of the CDS score (points)*					
Men		≤ 37 points	>37 - <55	≥ 55 points	
Women		≤ 34 points	>34 - <51	≥ 51 points	
*Socio demographic factors*					
Female, n (%)	674 (75.7)	229 (75.6)	213 (75.5)	232 (76.1)	0.986
Age in years, median (IQR)	84 (79,88)	85 (79,89)	84 (79,88)	84 (79,88)	0.180**
Length of stay on ward in months, median (IQR)	18 (5,40)	31 (11,58)	17 (3,34)	12 (3,31)	<0.001**
CDS: median (IQR)	44 (30,56)	26 (21,31)	44 (39,48)	60 (55,64)	NA
Cranberry use	443 (49.8)	155 (51.2)	135 (47.9)	153 (50.2)	0.720
*Comorbidities n (%)*					
Myocardial infarction	78 (8.8)^$^	28 (9.3)	28 (10.0)	22 (7.3)	0.482
Stroke	204 (23.1)^$^	79 (26.3)	63 (22.5)	62 (20.4)	0.215
Cancer	164 (18.7)^$^	49 (16.4)	49 (17.7)	66 (21.8)	0.209
Diabetes mellitus	174 (19.6)	54 (17.8)	54 (19.1)	66 (21.6)	0.484
COPD	129 (14.8)^$^	47 (15.8)	46 (16.5)	36 (12.1)	0.274
Urine incontinence	563 (65.8)^$^	263 (88.3)	180 (67.4)	120 (41.4)	<0.001
Urinary tract infection preceding year	386 (43.4)^$^	136 (44.9)	120 (42.6)	130 (42.8)	0.817
Dementia	677 (76.8)^$^	262 (87.3)	224 (80.6)	191 (63.0)	<0.001

CDS = Care Dependency Scale (range 15–75 points); IQR = interquartile range; COPD = chronic obstructive pulmonary disease; NA = not applicable.

^#^Low CDS group = most dependent on care; High CDS group = least dependent on care; ^$^n = 1-17 missing; *Chi-square test; **Kruskal-Wallis test.

There was no significant difference in age between the CDS groups (Kruskal-Wallis test; *P* = 0.180). The CDS score was negatively correlated with the length of stay on the ward: participants who stayed the longest had the lowest CDS scores (Kruskal-Wallis test; *P* < 0.001). There were no significant differences between the CDS groups for cranberry use, myocardial infarction, stroke, cancer, diabetes mellitus, COPD and urinary tract infection in the preceding year. However, urine incontinence and dementia were more frequently present in the low CDS group compared with the other groups (Table 1).

### Care dependency and mortality

**Figure 1:**
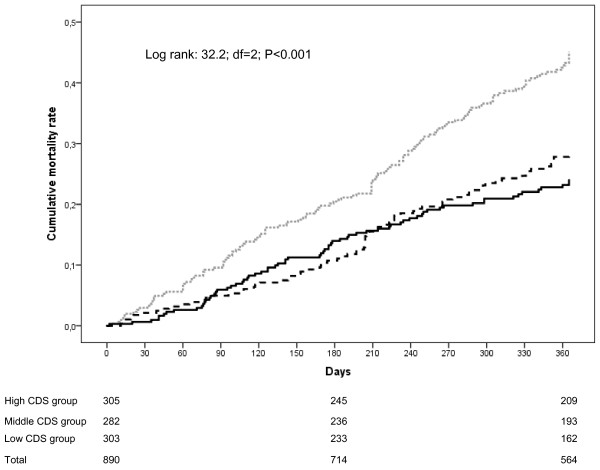
**Cumulative mortality rate depending on care dependency scores at baseline during 12-months of follow-up.** Grey dotted line: low CDS-group; Black dotted line: middle CDS-group; Solid black line: high CDS-group.

Figure 1 presents the mortality rate for the three CDS groups; the highest mortality rate was in the group with the lowest CDS score (log rank 32.2; df = 2; *P for trend* <0.001).

The mortality risk at 12-month follow-up, based on continuous CDS scores at baseline are presented in Table 2. The crude analysis shows, that per point decrease in CDS score, the mortality risk increased with 2% (HR 1.02; 95% CI 1.01-1.03). The adjusted models showed similar results.

**Table 2:** Mortality risk at 12-month follow-up depending on care dependency scores (continuous) at baseline

	**Mortality risk per point decrease in CDS score**	**95% CI**
Crude	1.02	1.01-1.03
Adjusted for age and gender	1.02	1.01-1.03
Adjusted for age, gender, cranberry use and LTCF	1.02	1.01-1.03
Adjusted for age, gender, cranberry use, LTCF, and length of stay on ward	1.02	1.01-1.03
Adjusted for age, gender, cranberry use, LTCF, length of stay on the ward and comorbidity*	1.03	1.02-1.04
Adjusted for age, gender, length of stay, cranberry use, LTCF comorbidity* and dementia	1.03	1.02-1.04

CDS = Care Dependency Scale (range 15–75 points); LTCF = Long-term care facility.

Data are presented as risk per point decrease (hazard ratio and corresponding 95% confidence intervals); estimated by Cox regression analysis.

*Comorbidity (including myocardial infarction, stroke, cancer, diabetes mellitus, COPD, and urine incontinence, urinary tract infection preceding year).

Additional analysis showed that a one point decrease in CDS score between 0 and 6 months was related to an increased mortality risk of 4% during the subsequent 6 months follow-up, adjusted for baseline CDS score (HR 1.04; 95% CI 1.03-1.06).

### Change in care dependency during 12 months of follow-up

Table 3 shows the variation in care dependency among survivors for the CDS groups at 0–6 months (n = 714) and 7–12 months (n = 564) follow-up, based on the 33% gender-specific CDS score at baseline for the first 6 months and 33% gender-specific CDS score at 6-month follow-up. The pattern of ‘improvement’, ‘being stable’ and ‘degradation’ of care dependency was almost similar over the two 6-month periods.

**Table 3:** Variation in care dependency in survivors during 2 subsequent periods of 6 months of follow-up for the three CDS groups

	**Low CDS group**	**Middle CDS group**	**High CDS group**	**Total group**
*0-6 months: n (%)*				
n	233	236	245	714
Improved	41 (17.6)	30 (12.7)	--	71 (9.9)
Stable	192 (82.4)	120 (50.8)	155 (63.3)	467 (65.6)
Deterioration	--	86 (36.4)	90 (36.7)	176 (24.6)
*7-12 months: n (%)*				
n	210	184	170	564
Improved	42 (20.0)	22 (12.0)	--	64 (11.3)
Stable	168 (80.0)	98 (53.3)	109 (64.1)	375 (66.5)
Deterioration	--	64 (34.8)	61 (35.9)	125 (22.2)

CDS = Care Dependency Scale (range 15–75 points).

### Predictive factors for increase in care dependency

Table 4 presents the results of the crude and adjusted multivariate linear regression analysis at 6 months. The adjusted model at 6 months showed that gender, age, baseline CDS score, cancer, urine incontinence and dementia predicted an accelerated decrease of dependency scores at 6 months. Cranberry use, LTCF, myocardial infarction, stroke, diabetes mellitus, COPD, and urinary tract infection in the preceding year, were not associated with the CDS score at 6 months. We did not find co-linearity between the dependent variable (CDS-score at 6 months) and the independent variables in both the crude and adjusted model. The Variance Inflation Factors ranges between 1.0 and 1.4. The multivariate linear regression model for men and women separately showed similar results (data not shown).

**Table 4:** Predictors of the care dependency score for survivors at 6-month follow-up (n = 659)

	Crude model*			Adjusted model**		
	**B**	**SE**	***P*-value**	**B**	**SE**	***P*-value**
Constant	22.37	5.18	<0.001	22.73	4.92	<0.001
Female	1.839	0.96	0.056	1.854	0.92	0.045
Age in years	-0.106	0.06	0.058	-0.122	0.06	0.027
Cranberry use	0.143	0.78	0.855			
Long-term care facility	-0.079	0.04	0.055	-0.075	0.04	0.063
Length of stay on ward in months	-0.009	0.01	0.486			
Baseline CDS score	0.685	0.03	<0.001	0.693	0.03	<0.001
Myocardial infarction	-0.819	1.38	0.552			
Stroke	-0.539	0.96	0.576			
Cancer	-2.927	1.01	0.004	-2.969	1.00	0.003
Diabetes mellitus	0.163	0.99	0.869			
COPD	0.756	1.13	0.497			
Urine incontinence	-3.109	0.93	0.001	-3.171	0.92	0.001
Urinary tract infection preceding year	-0.634	0.81	0.432			
Dementia	-3.779	1.03	<0.001	-3.543	0.97	<0.001

CDS = Care Dependency Scale (range 15–75 points); SE = Standard Error; COPD = Chronic obstructive pulmonary disease.

*multivariate linear regression model.

**excluded from the model: cranberry use, myocardial infarction, stroke, diabetes mellitus, COPD, urinary tract infection preceding year.

## Discussion

The main purpose of this study was to gain insight in the stability and changes in the care dependency status of LTCF residents, to explore possible predictive factors of change in care dependency, and examine the effect of care dependency on mortality. Changes in care dependency were examined to shed light on how to manage care and provide better tailored care for individual LTCF residents.

### Care dependency and mortality

In studying the natural course of care dependency, the relation between care dependency and mortality is important. It can be hypothesized that higher care dependency leads to higher mortality risk. There are a few studies unraveling this relation. The study of Marengoni et al. showed that baseline disability was a strong predictor for mortality, independent of number of diseases [25]. Also Chen et al. showed that the sum of care problems, independent of comorbidity, is a predictor of 12-month mortality in LTCF residents [26], and Ferrucci et al. concluded that mortality after severe disability onset was high [27]. Within our study, we found similar results. A one point decrease in baseline CDS score was related to a 2% higher mortality risk in the forthcoming 12-months, also when adjusting for age, gender, cranberry use, LTCF, length of stay on the ward, comorbidity and dementia.

### The course of care dependency

A recent Swiss study among 10,199 nursing home residents (70% women, 74% aged 80 years and above) observed a decrease in activities of daily living (ADL) of 35% and an increase in ADL of almost 14% among residents, within a period of median 6 months (SD 3 months) [12]. They used the Minimum Data Set Activities of Daily Living (MDS-ADL) and looked at ADL performance as primary outcome. Another study in low ADL-dependent LTCF residents in the USA, found that 69% of these LTCF residents with higher physical function remained stable in their ADL performance during 6 months of follow-up [13]. Our study shows a similar trend for care dependency. The majority of the LTCF residents remained stable in their care dependency status, only 10% improved and 25% deteriorated. The variability in the pattern of ‘improvement’, ‘being stable’ and ‘degradation’ of care dependency varies in a similar pattern over two subsequent 6-month periods.

### Predictors of change in care dependency

It is known that LTCF residents with cognitive impairment experience a deterioration in their ADL performance [12,13,15], which make them increasingly dependent on nursing care.

As mentioned earlier, nutritional status [12,13], cognitive impairment [12,13,15], absence of daily contact with proxies [12], depression [12,16], neuropsychological deficits [17], incontinence [12,13,18] and infections [19] were mentioned as predictors for deterioration in ADL performance. The study of Dijkstra et al. showed that the degree of care dependency at entry to the study was one of the strongest predictors of follow-up CDS ratings [14]. Our study confirms that the baseline CDS score is predictive, but showed also that gender, age, urine incontinence, dementia and cancer; predict an increase in care dependency over time.

### Strengths and limitations

The present study included a large sample of 890 residents residing in 21 Dutch LTCFs. Our study participants represent a vulnerable population; with a median age of 84 years and a high dependency on nursing care (median CDS score of 44 points). This median baseline CDS score is comparable with that of other studies in nursing homes [11,28]. Because a recent international comparison of the CDS demonstrated its usefulness for comparative research across countries [28], the results of the present study might be generalizable to LTCFs worldwide.

Daily nursing care in LTCF focuses on residents care dependency as a process in which the residents’ self-care decreases, and in which care demands makes a person increasingly dependent on nursing care [7]. Although other instruments to assess care dependency are available (e.g. the MDS-ADL, Barthel index [29], or Katz [30]) we decided to use the Care Dependency Scale. The CDS comprises all domains of nursing care; it is not limited to basic ADL, but also includes the individual’s capacity for social contacts, recreational activities, and learning abilities. The CDS is easy to administer, the responsible nurse could assess the CDS usually in less than five minutes and has shown satisfactory reliability and validity [22-24].

Our study was nested in the CRANBERRY trial. Since the CRANBERRY study is a randomized-controlled trial and half of the participants underwent treatment with cranberry, this could have influenced the course of care dependency. However, there was no cranberry effect on care dependency over time. Therefore the CRANBERRY trial gives us the possibility to explore whether there are predictive factors of changes in care dependency. However, this means that not all earlier mentioned predictors of change in ADL were included in the dataset.

Within this study we were particularly interested to explore the personal characteristics of LTCF residents on the natural course of care dependency. An institutional effect on mortality and care dependency was not found. Other factors dependent on organizational characteristics of the long-term care facilities would be of interest for further research, since these characteristics could influence the care dependency status of LTCF residents as well. However, this was outside the scope of our study.

Another possible limitation of the present study is that we studied a selected period of 12 months. Classification of the participants into the three CDS groups was based on the prevalent CDS score at baseline, and we have no data on the CDS score of the residents at admission to the LTCF. Because care dependency is a dynamic process, the change in CDS score (and therefore the results) might be different if we had known the care dependency status when the residents were first admitted.

### Implications for practice

A regular and simple assessment of care dependency can be valuable, since this allows nursing staff to become more aware of the variability in the care dependency status of their residents, manage care, and provide better tailored care for individual residents. In daily nursing care, they are the first professionals who might observe subtle changes in the care dependency status of residents and therefore can better anticipate residents’ care needs. The present study shows that residents can increase or as well as decrease in their level of care dependency. Care dependency may be influenced by individually tailored interventions and this needs further exploration in research. In addition, in view of the association between the CDS score and mortality, it seems relevant to train staff in providing palliative care as well as restorative care [31].

## Conclusions

The majority of surviving LTCF residents were stable in their care dependency status over two subsequent 6-month periods, even 10% showed improvement and 25% deteriorated in their dependency status. Highly care dependent residents showed an increased mortality risk. Awareness of the natural course of care dependency is essential to residents and their formal and informal caregivers when considering therapeutic and end-of-life care options.

## Abbreviations

ADL: Activities of daily living; CDS: Care dependency scale; CI: Confidence interval; COPD: Chronic obstructive pulmonary disease; HR: Hazard ratio; IQR: Interquartile range; LTCF: Long-term care facility; NTR: Dutch trial register.

## Competing interest

All researchers worked independently from the funders. The authors declare that they have no competing interests.

## Authors’ contributions

MAAC, HJMC and JG contributed to the study concept and design, acquisition of data, analysis and interpretation of the data, drafting of the manuscript and critical revision of the manuscript. All authors read and approved the final version of the manuscript.

## Pre-publication history

The pre-publication history for this paper can be accessed here:

http://www.biomedcentral.com/1471-2318/14/67/prepub
